# Green synthesis of low-cost graphene oxide-nano zerovalent iron composite from solid waste for photocatalytic removal of antibiotics

**DOI:** 10.1016/j.isci.2024.111486

**Published:** 2024-11-28

**Authors:** Aditya Kumar Jha, Sukalyan Chakraborty, Jayanta Kumar Biswas

**Affiliations:** 1 Department of Civil and Environmental Engineering, Birla Institute of Technology, Mesra, Ranchi 835215, India; 2 Department of Civil Engineering, Cambridge Institute of Technology, Ranchi 835103, India; 3 Enviromicrobiology, Ecotoxicology and Ecotechnology Research Laboratory (3E-MicroToxTech Lab), Department of Ecological Studies, University of Kalyani, Kalyani, Nadia 741235 West Bengal, India; 4 International Centre for Ecological Engineering, University of Kalyani, Kalyani 741235 West Bengal, India

**Keywords:** Catalysis, Aquatic science, Aquatic biology

## Abstract

This study develops a graphene oxide-nano zerovalent iron (GO-nZVI) composite for the efficient removal of tetracycline and ciprofloxacin from water. The composite was synthesized using sugarcane bagasse as the matrix for graphene oxide (GO) and Sal leaf extract to reduce iron into nano zerovalent iron (nZVI). Microscopic analysis confirmed multiple GO layers with nZVI particles on their surface, while XRD and Raman spectroscopy verified the crystalline nature of the composite. Photocatalytic degradation achieved removal efficiencies of 91% for tetracycline and 92% for ciprofloxacin. The microbial assays showed that the degraded antibiotics were non-toxic, ensuring their safe disposal. Treatment costs were estimated at 53 USD for tetracycline and 68 USD for ciprofloxacin per 10,000 L of contaminated water. This approach provides a sustainable solution by employing agricultural waste in environmental remediation, supporting a circular economy model for tackling antibiotic contamination in water.

## Highlights

• The study targeted antibiotic removal from water using GO-nZVI composite
• GO was derived from bagasse; nZVI was synthesized via the Sal leaf extract green route
• High antibiotic degradation efficiency made them non-toxic to bacterial strains
• Economic analysis confirmed the sustainability of the synthesis and treatment

## Graphical abstract

**Figure:**
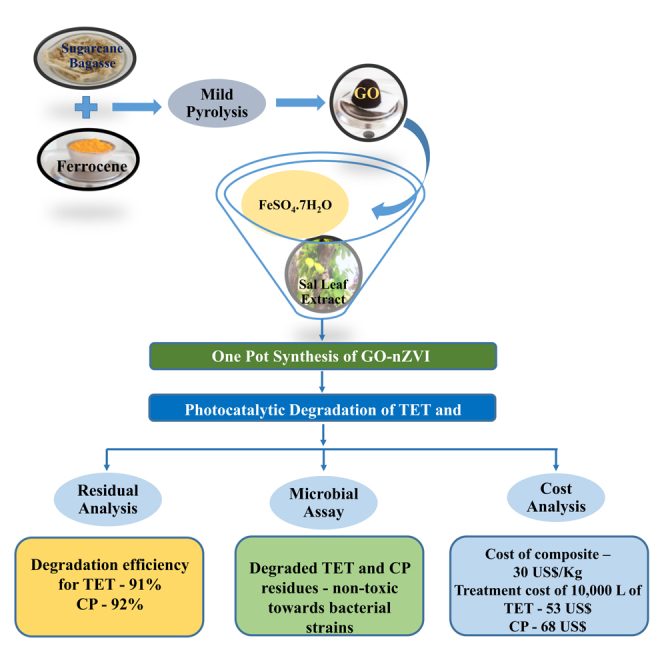

## Introduction

Antibiotics help to fight bacterial infections in human beings, the livestock industry, and aquaculture.^1^ However, only a fraction of the antibiotics extensively used for therapy are absorbed by the life forms, and the majority are discharged into the environment, causing ubiquitous antibiotic contamination.^2^ Further, though the half-life of antibiotics is short, the breakdown residues are persistent.^3^ These antibiotic residues are toxic, mutagenic and carcinogenic to aquatic animals.^4^^,^^5^ These residues can even enter the food chain and ultimately reach the human body,^6^ leading to disturbance of the human microbiome along with antibiotic resistance, treatment failure, and mortality.^3^^,^^4^ So, an urgent need arises to treat these effluents from water to avoid their detrimental environmental effects. Researchers worldwide have tried developing numerous scientific solutions to mitigate this problem, but treatment methods exhibited several drawbacks and limitations.^7^^,^^8^ To overcome the drawbacks, emerging technologies are required for enhanced removal of residual antibiotics.

In the last decade, the application of nanotechnology for pollutant removal has been quite successful, with considerably high efficiency. Recently, nZVI-based materials have proven excellent photocatalysts against dyes, antibiotics, and other organic pollutants.^7^^,^^8^ Though their conventional synthesis posed specific environmental issues, biochemical synthesis has been championed as the most sustainable, economical, and safe method of nZVI synthesis. Green synthesis naturally eliminates the application of unsafe chemicals and intense temperatures and has a scope of extensive synthesis.^7^

A search on 19th April 2023 on the Web of Science with keywords “photocatalytic degradation antibiotics nano” in the topic field (link: photocatalytic degradation antibiotics nano (Topic) – 276 – Web of Science Core Collection) yielded 276 articles, which included 261 research articles and 15 review articles. The first article was published in 2010, and exponential growth in the number of publications was observed, with around 84% of the articles published in the last five years. A detailed trend analysis of this topic is supplied as supplementary material (Figure S3, Tables S1 and S2).

However, the application of bare nZVI has some shortcomings, such as agglomeration, rapid oxidation in water and air, poor selectivity, poor dispersibility, and insufficient mass transfer at the catalyst-solution interface, which can cause surface passivation, hampering its dispersion and functionalities.^9^ Separation of nZVI from wastewater after treatment is also problematic. Fortunately, incorporating nZVI on a composite with porous solid materials having sufficient cracks and pores can help tackle this problem. Among various composites, GO-based composites have served as an effective and safe material for treating pollutants. Graphene oxide-based composites are environmentally safe, economical, and highly efficient photocatalysts with several unique properties. In addition, the C=C bond leads to π-π interaction, C=O leads to hydrogen bonding, while the –OH group enhances cation exchange.^10^

Thus, in this study, a detailed investigation was undertaken to develop useful materials from waste materials to effectively treat two selected antibiotic residues (Tetracycline and Ciprofloxacin) in the aqueous phase with environmental parameter optimization and potential practical application evaluation. Tetracycline (TET) is an extensively used, broad-spectrum antibiotic that effectively treats bacterial and fungal infections in humans, livestock, and aquaculture. Ciprofloxacin (CP), the fluoroquinolone class of antibiotics, is prescribed for urinary tract, respiratory tract, skin, bone, soft tissue infection, and tuberculosis. It is one of the most common antibiotics present in hospital wastewater in significant concentration,^11^ and in India, up to 31 mgL^-1^ of CP has been detected in sewage.^12^

Utilizing readily available, eco-friendly agro-waste for synthesizing nano-catalysts and their application in removing antibiotic residue from water can be promising in safeguarding the environment. This is because degradable agro-waste constitutes a substantial volume of solid waste, which, when dumped into landfills or open dumping sites, leads to negative environmental consequences such as air pollution from produced gases, emission of greenhouse gases, and water contamination from the leachate seeping into the groundwater and polluting the soil.

The prevailing socio-economic model till now was based on how products are produced, consumed, and disposed of. The linear economy relied on the “take-make-dispose” cycle where raw materials are collected and transformed into products, utilized, and disposed of. But, with the increasing global population and limited availability of raw materials, the utilization of waste material of one product as raw material for other products has become a necessity. To address this challenge, the circular economy and nexus thinking principles have gained attention, aiming to reduce, reuse, recycle, and recover materials. They are considered crucial for achieving sustainability objectives and a circular economy. Considering these facts, the waste biomass of Sugarcane bagasse, an agro waste rich in cellulose, was chosen as the base material considering a circular economic approach, where solid waste is converted to a value-added material for wastewater management, addressing the Sustainability Development Goals 6 and 15.

## Results and discussion

### Synthesis and characterization of graphene oxide-nano zerovalent iron composite

The synthesis of the GO-nZVI composite took place initially in colloidal form, which was then filtered and dried. The parameters for the synthesis were optimized based on the degradation efficiency of the formed composite in 20 mgL^-1^ CP solution. The degradation efficiency increased with the incremental volume of FeSO_4_.7H_2_O and Sal leaf extract in GO up to a certain level and then decreased. The optimum degradation of CP was achieved by a composite created with 100 mL FeSO_4_.7H_2_O solution, 0.1 g of GO, and 100 mL of Sal leaf extract (Figure S2).

**Figure 1:**
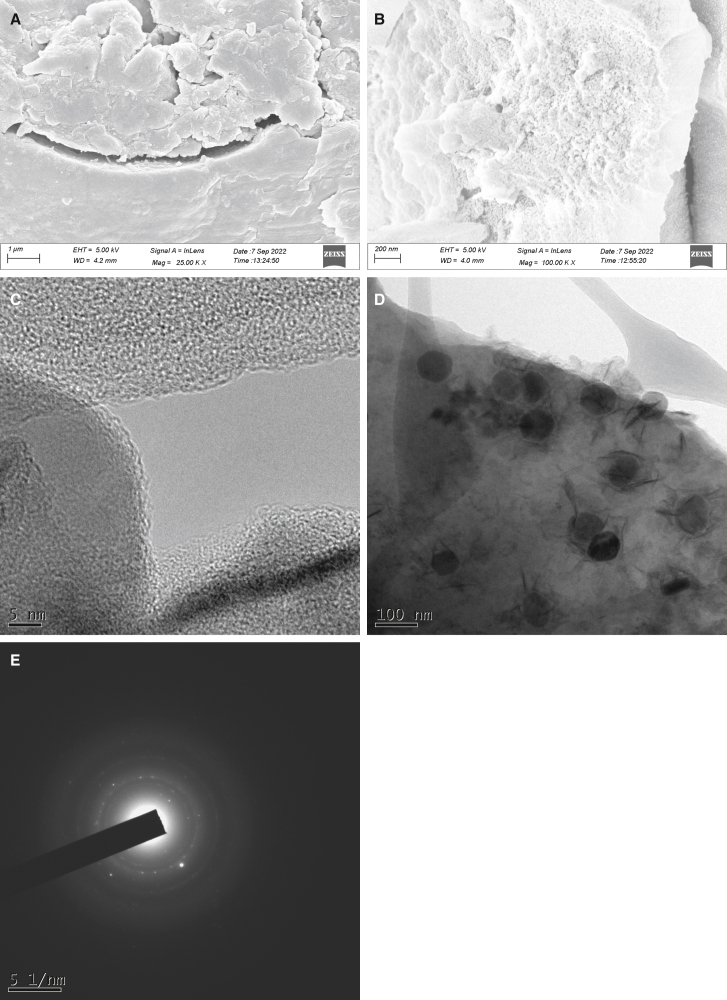
Morphological characteristics of the synthesized composite FESEM of composite at magnification (A) 25000, and (B) 100000; HRTEM of composite (C) showing layers of GO, and (D) nZVI particles present on the surface of composite; and (E) SAED image of GO-nZVI composite showing bright spots and rings indicating its crystalline nature.

This synthesized material was further characterized for the validation of the material. The surface morphology of the composite was examined by FESEM and HRTEM. The FESEM image at 25,000X magnification showed aggregated and wrinkled layers/sheets randomly overlapping, a characteristic of GO (Figures 1A), S6). The FESEM image at magnification 100,000X showed spherical aggregated particles, apparently in the nano-range (Figure 1A). The HRTEM image of the composite at very high magnification clearly showed the overlapping layers of GO, with spherical nZVI particles on top of it (Figures 1C and 1D). The size of the nZVI determined using ImageJ software, varied from 43 nm to 85 nm with an average length of 79 nm (standard deviation = 12 nm; median = 66 nm). Morphological observation of wrinkles and uneven surfaces of the composite suggest enhanced surface area and good photocatalytic efficiency due to the present active sites.^13^ Transparency in the composite layers indicates the synthesis of few-layered GO, with high photo-absorption and high photocatalytic efficiency against pollutants.^14^ Bright spots and rings in the SAED image (Figure 1E) indicated the crystalline nature of the particles formed.

**Figure 2:**
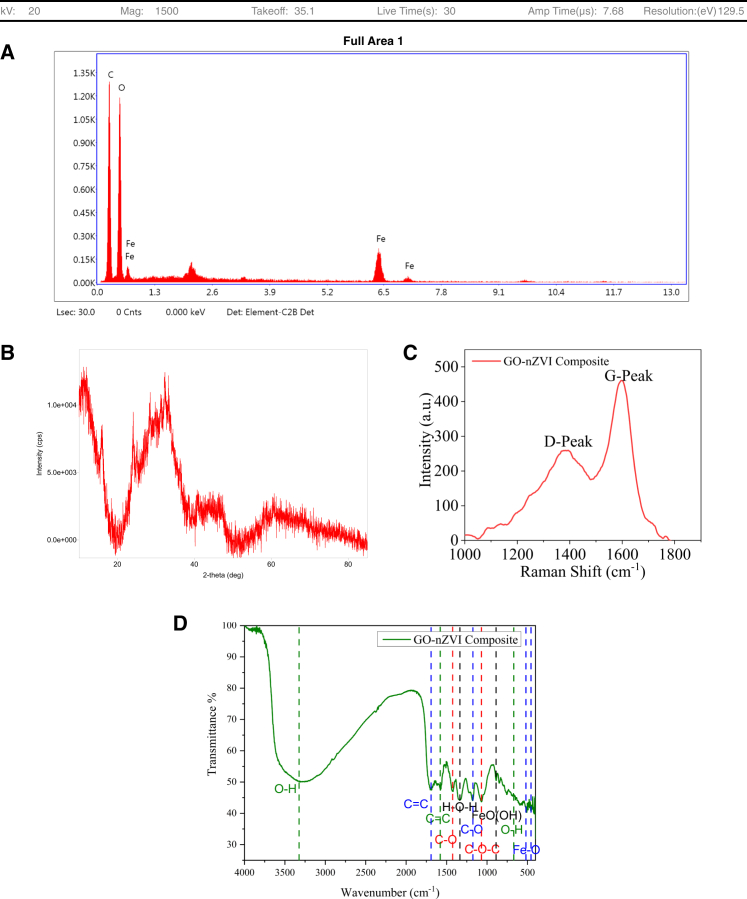
Composition, Functional groups and purity of the synthesized composite The surface morphology, crystallinity, elemental composition, and purity of the synthesized GO-nZVI particles are presented in the image (A) EDS, (B) XRD, (C) RAMAN, and (D) FTIR image of the GO-nZVI composite.

The elemental composition of the synthesized composite using EDX showed oxygen to be the most abundant element (44.7%) by mass, followed by carbon (38.8%) and iron (16.5%) (Figure 2A). The source of oxygen and carbon can be sugarcane bagasse, while the presence of Fe can be attributed to FeSO_4_.7H_2_O. The XRD analysis of the composite showed peaks at 11.6°, 44.6°, and 64.8° (Figure 2B). The peak at 11.6° indicates the presence of GO in the composite,^14^ while the peak at 44.6° and 64.8° shows the presence of Fe^0^ in the composite.^15^ Apart from this, a peak in the range of 30°–35° is also present, which can be due to the presence of oxidized forms of iron Fe_2_O_3_, FeO(OH), and Fe_3_O_4_.^16^^,^^17^

The Raman spectroscopy showed peaks at 1358 and 1585 cm^−1^, corresponding to the characteristic D and G peaks of GO, respectively. The G-peak is characterized by the E2g vibrations created by sp2 vibrations of carbon present in the hexagonal carbon network. In contrast, the D-peak is made by the disorders present in the GO’s structure.^18^ The intensity of D-peak to the intensity of G-peak (I-D/I-G) determines the degree of oxidation and defects. Since I-D is less than I-G, the GO present in the composite has low defects and high purity.^19^ The FTIR of the synthesized composite showed peaks at 3300 cm^−1^, which could be attributed to O-H, 1637 cm^−1^ owing to C=C,^20^ 1572 cm^−1^ owing to C=C stretching vibrations,^10^ 1426 cm^−1^ owing to C-O carboxyl bond,^14^ 1351 cm^−1^ owing to hydrogen bond stretching,^21^ 1182 cm^−1^ owing to C-O stretching vibration,^20^ 1076 cm^−1^ is owing to the C-*O*-C asymmetric stretching of GO groups,^21^^,^^22^ 895 cm^−1^ is owing to FeO(OH),^23^ 689 cm^−1^ is due to O-H stretching vibrations,^21^ 571 cm^−1^ and 462 cm^−1^ is owing to Fe-O group of Fe_3_O_4_^10^ (Figure 2D).

### Degradation study of tetracycline and ciprofloxacin

**Figure 3:**
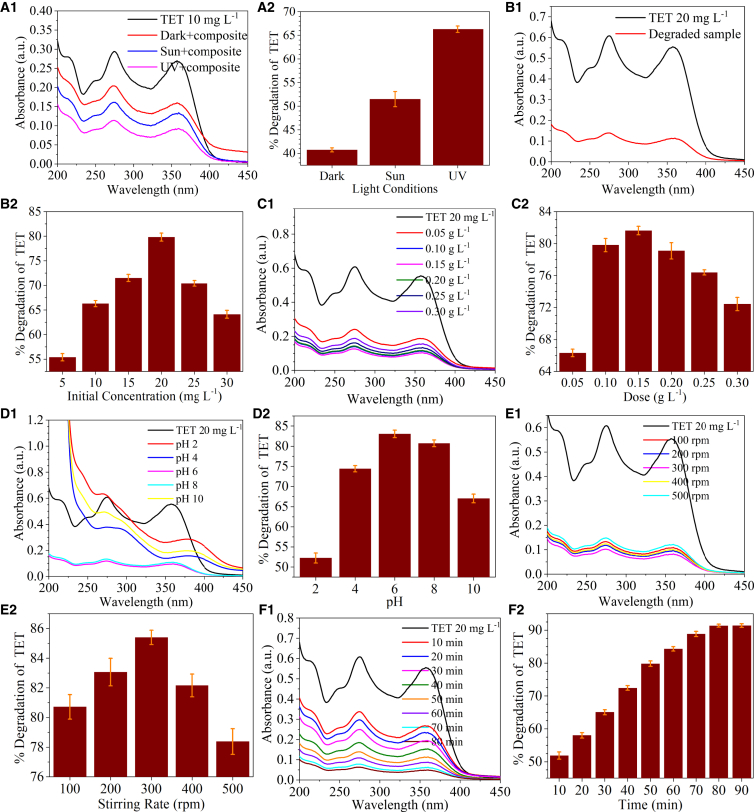
The parameter optimization for the degradation process of the antibiotic, tetracycline with the synthesized GO-nZVI particles are presented in the figure as (a1, b1, c1, d1, e1, and f1) for the optimization of the environmental variables and (a2, b2, c2, d2, e2, and f2) for degradation

**Figure 4:**
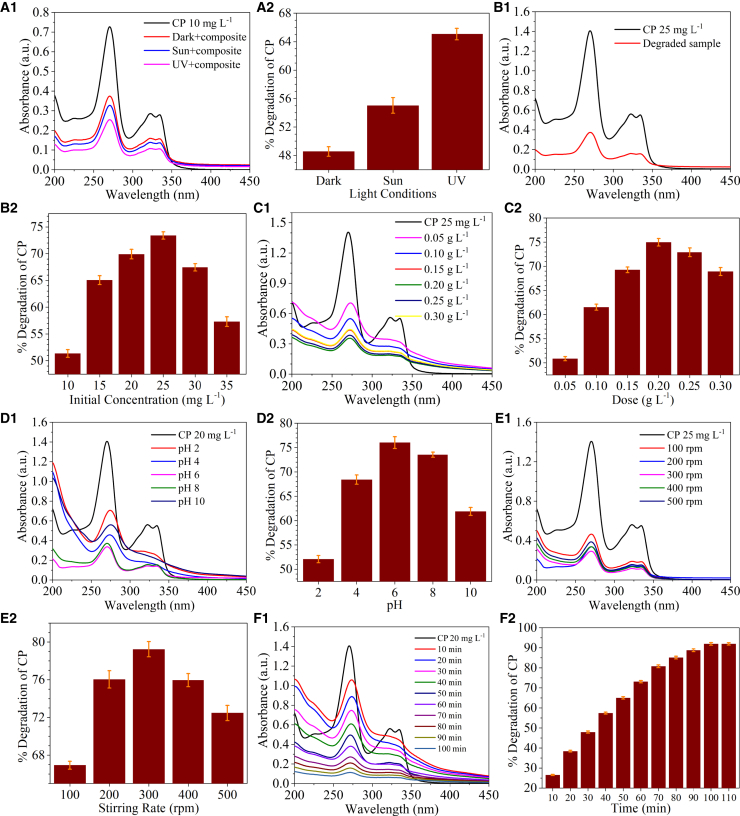
The parameter optimization for the degradation process of the antibiotic, Ciprofloxacin with the synthesized GO-nZVI particles are presented in the figure as (a1, b1, c1, d1, e1, and f1) for the optimization of the environmental variables and (a2, b2, c2, d2, e2, and f2) for degradation

Light has a massive role in photocatalytic degradation.^24^ In this study, the photocatalytic degradation was performed under sunlight and UV light, while a control experiment was set up in the dark. The maximum removal efficiency of 66.2% was achieved in UV light, followed by 51.4% in sunlight and 40.7% in the dark, in the case of TET (Figure 3a1 and 3a2). Similarly, for CP, the maximum removal efficiency of 65.0%, 55.0%, and 48.5% were obtained in UV light, sunlight, and dark, respectively. Thus, further degradation studies were conducted in UV light (Figure 4a1 and a2).

The initial concentrations of TET and CP were considered 5, 10, 15, 20, 25, 30, and 35 mgL^-1^, considering the maximum concentration of the antibiotics found in the Indian context. Varied degradation efficiency was achieved for TET (55, 66, 71, 79, 70, and 64%) and CP (51, 65, 69, 73, 67, and 57%) at 5, 10, 15, 20, 25, 30, and 35 mgL^-1^, respectively. With an increase in initial concentration from 10 mgL^-1^ to 20 mgL^-1^, the degradation efficiency increased and then decreased to 35 mgL^-1^ (Figure 3b1 and 3b2). For CP, the increase in initial concentration from 10 mgL^-1^ to 25 mgL^-1^ led to an increase in the degradation efficiency, and then with further increase in concentration up to 35 mgL^-1^ showed a decrease in efficiency (Figure 4b1 and b2). The increase in the antibiotic concentration leads to a higher number of antibiotic molecules present in the solution, thereby increasing the probability of interaction with the photocatalyst molecules. Hence, the degradation efficiency initially increases with the increase in antibiotic concentration until the equilibrium is achieved.^25^ Once the antibiotic concentration is increased beyond this point, the number of photocatalyst molecules is insufficient to interact with the increased number of antibiotic molecules and their intermediates, thus decreasing the degradation efficiency.^26^^,^^27^

This was followed by variation in dose of GO-nZVI composite as 0.05, 0.10, 0.15, 0.20, 0.25, and 0.30 gL^-1^. As the dose was increased from 0.05 to 0.30 gL^-1^, the degradation efficiency of TET varied as 66, 79, 81, 79, 76, and 72%, whereas the increasing order of variation for CP was 50, 61, 69, 74, 72, and 68.9% respectively. TET’s degradation efficiency increased with an increase in GO-nZVI composite dose up to 0.15 gL^-1^. However, with a further increase in dose up to 0.30 gL^-1^, the degradation efficiency decreased (Figure 3c1 and c2). The degradation efficiency of CP showed a similar trend, with an improvement from 50% at 0.05 gL^-1^ to 74% at 0.20 gL^-1^. However, a decline in the degradation efficiency to 68% was observed with a further increase in dose up to 0.30 gL^-1^ (Figure 4c1 and c2). The increase in degradation efficiency with an increase in GO-nZVI composite dose could be due to an increase in active sites and reactive species. This leads to enhanced interaction with the antibiotic molecules and their intermediates, hence improved degradation.^28^ Beyond the optimum level of catalyst dose, the penetration of UV light may reduce, and the scattering of light can increase. The chances of the agglomeration of the photocatalyst also increase at higher concentrations. All these factors ultimately dampen the degradation efficiency of antibiotics.^25^

The pH of the antibiotic solutions varied from 2 to 10. In the case of TET, the degradation efficiency increased gradually with an increase in pH from 2 to 6 (52% at pH 2; 74% at pH 4; 83% at pH 6), followed by a gradual decline after that (80% at pH 8; 67% at pH 10) (Figure 3d1 and 3d2). CP demonstrated a similar trend of degradation efficiency with a sequential increase in pH (Figure 4d1 and d2). The pH of antibiotic solution plays a significant role in generating active species and radicals and determines the degradation pathway and structure of fragmented compounds.^29^ At a very low pH, H+ ions are readily available in the solution, which can consume OH- and –OH, responsible for antibiotic degradation.^30^ Thus, the degradation efficiency at pH 2 was very low for both TET and CP; with increased pH, H+ concentration decreases, increasing the OH- and –OH concentration and, consecutively, the degradation efficiency. While at alkaline pH, a passive film forms over the surface of nZVI, which inhibits the interaction of nZVI with the antibiotic molecules, thus reducing the degradation efficiency at higher pH.^31^ The pHzpc of GO is around 3.9^32^ and nZVI is around 8.^33^ TET can act as a cation, zwitterion, and anion at pKa values of 3.3., 7.7, and 9.7, respectively.^8^ Similarly, CP can behave as a cation, neutral, and anion due to its pKa values of 6.0 and 8.6.^32^ At higher pH, TET, CP, and GO-nZVI have a negative charge, resulting in repulsion, reduced interaction, and diminished degradation efficacy^25^^,^^34^. At pH 6, TET, CP, and GO-nZVI composite have a neutral charge, thus allowing for good interaction between antibiotics and photocatalysts. Further studies were performed at pH 6.

The rate of stirring was optimized between 100 rpm and 500 rpm. Stirring can increase the dissolved oxygen in the solution and improve the production of superoxide radicals while enhancing the interaction of antibiotic molecules and photocatalysts.^35^^,^^36^ Maximum efficiency obtained for TET (Figure 3e1 and e2) and CP (Figure 4e1 and e2) were 85% and 79% respectively at 300 rpm.

**Figure 5:**
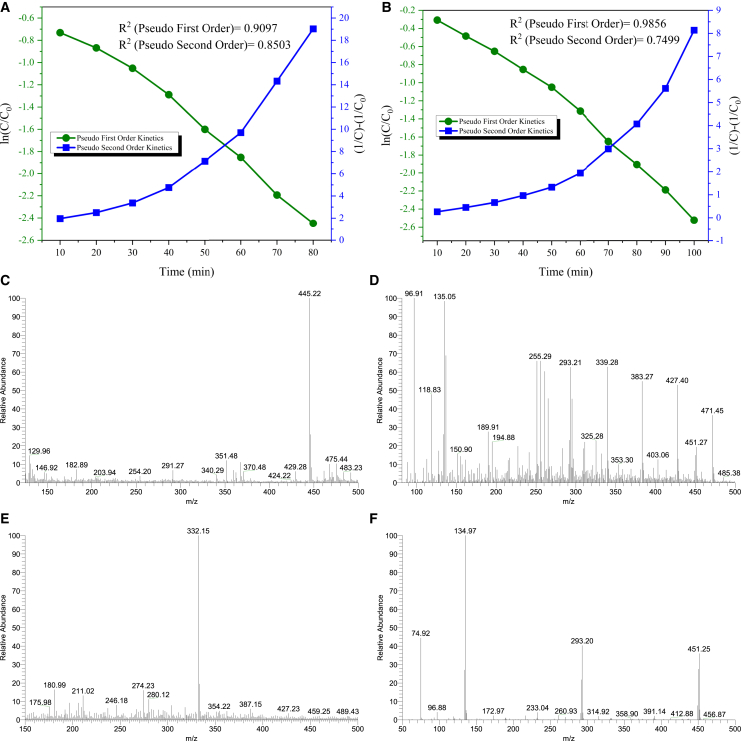
Kinetics and Byproduct Analysis of Antibiotic Degradation via Photocatalysis Reaction kinetics for the photocatalytic degradation of (A) TET, and (B) CP using GO-nZVI composite. Mass spectroscopy of (C) TET, (D) degraded TET solution, (E) CP, and degraded CP solution.

To find the optimum duration for the maximum degradation, the residual analysis of the antibiotics was done with the other optimized parameters every 10 min. TET showed a maximum degradation of 91% at 80 min (Figure 3f1 and 3f2), while CP showed a maximum degradation of 92% at 100 min (Figure 4f1, f2). Degradation kinetics was computed using the degradation results of TET and CP. For TET, the maximum degradation was achieved in UV light with 20 mgL^-1^ TET concentration, 0.15 gL^-1^ GO-nZVI composite dose, at pH 6, in 300 rpm stirring condition, in 80 min. For CP, the maximum degradation was achieved in UV light with 25 mgL^-1^ CP concentration, 0.2 gL^−1^ GO-nZVI composite dose, at pH 6, in 300 rpm stirring, in 100 min. The results were plotted in Equations 2 and 3 to determine the reaction rate. For TET, R^2^ for the pseudo-first-order reaction was 0.90, and for a pseudo-second-order response was 0.85 (Figure 5A). For CP, R^2^ for the pseudo-first-order reaction was 0.98, and for the pseudo-second-order response was 0.74 (Figure 5B). Both TET and CP followed the pseudo-first-order reaction. The rate constant (k), computed using the slope of the straight line, was 0.0318 min^−1^ for TET and 0.0238 min^−1^ for CP.

### The probable mechanism of degradation

The synthesisedGO-nZVI composite consists of GO and nZVI. GO and nZVI contain various oxygenated functional groups that can interact with antibiotics and degrade them.^7^^,^^37^ In addition to this, FTIR analysis of the GO-nZVI composite affirmed the existence of functional groups, which lead to the adsorption of antibiotics on the surface of the composite with C=C, C=O bond, and –OH group attachment leading to π-π interaction, hydrogen bonding, and cation exchange respectively.^10^ This is followed by the initiation of photocatalytic degradation, wherein electrons are transferred from the valence to the conduction band in the presence of UV light.

The electrons in the conduction band of GO, react with oxygen in water to generate $O_{2}^{∙-}$ (superoxide ion). These $O_{2}^{∙-}$ react with $H^{+}$ in water to produce H_2_O_2_ (hydrogen peroxide), a strong oxidizing agent. H_2_O_2_ reacts further to produce ·OH (hydroxyl radical). Due to the transfer of electrons to the conduction band, holes are developed in the valence band, which results in the formation of H^+^ and OH^−^ (hydroxyl ion).^23^

**Figure 6:**
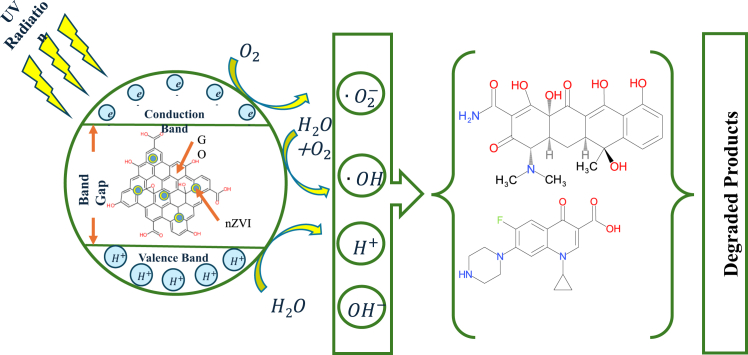
Probable mechanism of TET and CP degradation by the GO-nZVI composite

The nZVI has a core of Fe^0^ and an external shell of FeOOH. In an aqueous medium, Fe^0^ liberates e^−^ and transforms to Fe^2+^, which further transforms to Fe^3+^ by reacting with O_2_ and H^+^ and producing H_2_O_2_ in the process. The Fe^2+^ also reacts with O_2_ to produce $O_{2}^{∙-}$. H_2_O_2_ breaks into OH^−^ and ·OH. The ·OH, OH^−^, e^−^, $O_{2}^{∙-}$, and H^+^ generated in the process leads to the degradation of antibiotics.^29^^,^^38^ The spectrophotometric peak of TET and CP decreases over time, probably due to their degradation. Mass Spectroscopy of TET and CP suggest their successful degradation, as their peaks at 445 and 332 (m/z) respectively are absent in the degraded samples (Figure 6C–6F). The presence of new peaks in the degraded samples indicates the formation of breakdown products.

### Toxicity assay of degraded antibiotics

**Figure 7:**
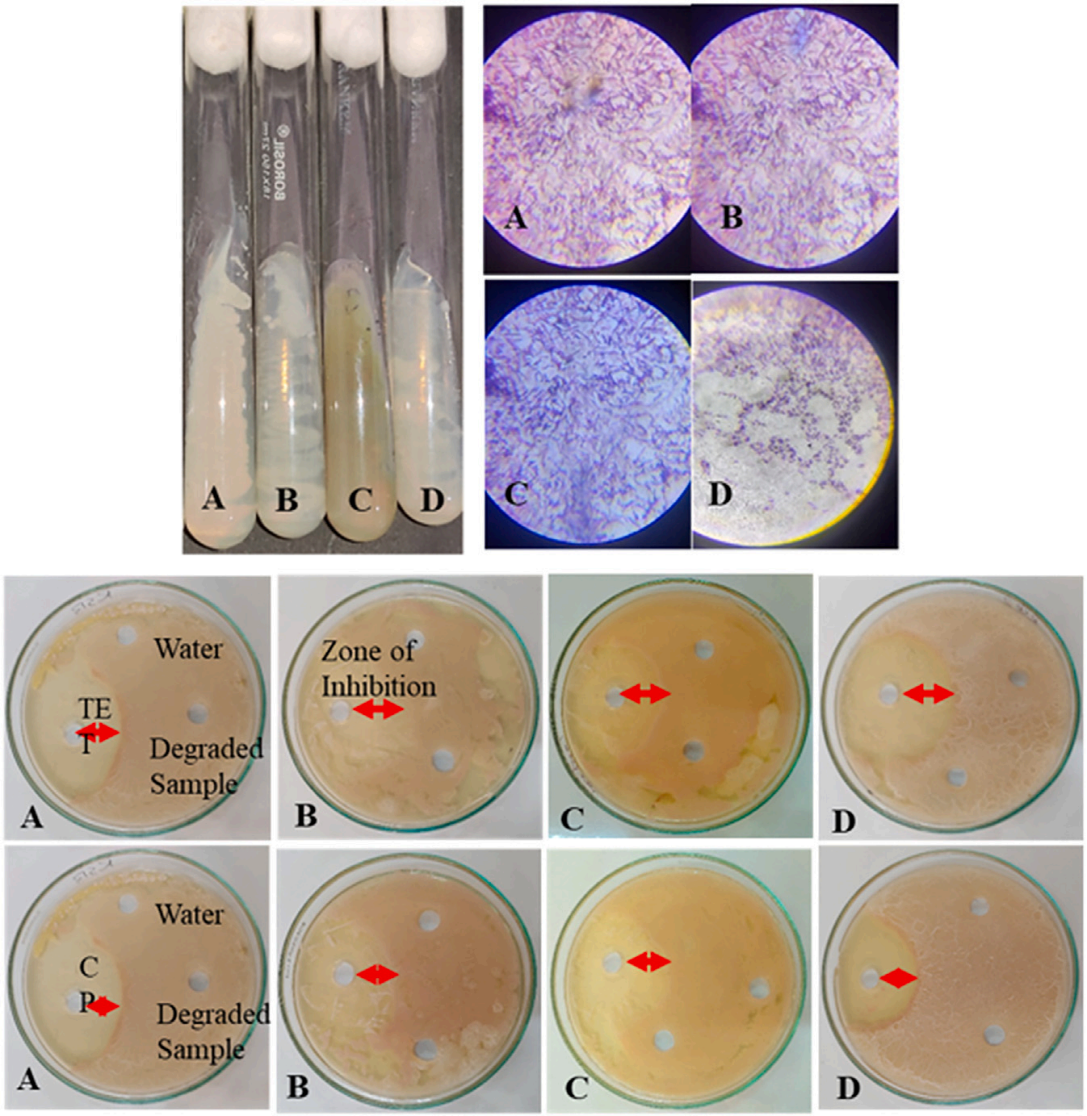
This image shows the isolated microbial strains, their gram-staining characters, and the zone of inhibition in the toxicity assay experiment with the degraded antibiotic solutions (A) Four distinct colony-forming species, namely A, B, C, and D, (B) gram-stained microscopic images of A, B, C, and D, respectively (C) toxicity assay of the degraded TET solution by A, B, C, and D (top 2) and degraded CP solution by A, B, C, and D (bottom 2).

The microbial toxicity assay performed on bacterial strains isolated from river Subarnarekha, as a representative of the natural waterbody where these degraded antibiotics can be discharged after treatment, exhibited satisfactory results. Four axenic bacterial strains (A, B, C, and D) were isolated with distinct morphological characteristics (Figure 7A). Gram staining revealed A, B, and D as Gram -ve and C as Gram +ve (Figure 7B). The toxicity assay revealed growth inhibition zones for all the bacterial strains around the well containing 20 mgL^-1^ CP and 25 mgL^-1^ TET (Figure 7C and 7D). However, no inhibition was observed around the wells containing the degraded samples of TET or CP. This observation suggests that the photo-catalytically degraded samples if discharged in natural waterbodies, would not pose any risk to the microbiome.

### Treatment cost analysis

To synthesize GO-nZVI composite, 1 g of GO required 1000 mL of FeSO_4_.7H_2_O and 1000 mL of leaf extract, yielding 2.5 g of composite. Electricity was needed to stir for 30 min and shake for 12 h. Thus, to produce 1000 g of composite, 400 g of GO, and 400 L of 0.01M FeSO_4_.7H_2_O was required. To synthesize 400 g of GO, 1080 g of sugarcane bagasse and 180.8 g of Ferrocene were needed. Electricity was consumed by the muffle furnace (2500W) during the pyrolysis process. So, combining all the prerequisite factors, the cost for synthesizing 1 kg GO-nZVI composite and degrading 10,000 L of TET/CP-laden wastewater was calculated as summarized in Table 1.

**Table 1:** Treatment cost of TET and CP

Items (1000 gramsGO-nZVI composite)		Rate	Required quantity		Cost
FeSO_4_.7H_2_O Ferrocene		750/Kg 8000/Kg	1200 g 180.8 g		900 (10.97 USD) 1447 (17.64 USD)
Electricity		7/KWh	14.7 KWh		103 (1.25 USD)
The total cost of synthesis of 1000 g GO-nZVI composite =					2450 (29.87 USD)
**Items (per 10,000 L TET and CP)**	**Rate**	**Requirements for TET**	**Price for TET**	**Requirements for CP**	**Price for CP**
GO-nZVI composite	2450 INR/29.87 USD per Kg	1500 g	3675 INR/44.81 USD	2000 g	4900 INR/59.75 USD
Electricity	7 INR/0.1 USD/KWh	100 KWH	700 INR/10 8.53 USD	100 KWH	700 INR/10 8.53 USD
Total price =			**4375 INR/53.3 USD**	Total price =	**5600 INR/68.29 USD**

### Conclusions

The present study successfully demonstrated the synthesis of a GO-nZVI composite capable of efficiently degrading two commonly used antibiotics, tetracycline and ciprofloxacin. The research identified the optimal environmental conditions for achieving the best degradation performance of the composite in aqueous solutions. Microbial assays on river-isolated bacteria exposed to the degraded antibiotics confirmed their conversion to completely harmless forms, ensuring safe discharge into the environment post-treatment. This study showcases a circular economic approach to simultaneously tackling solid waste disposal and wastewater treatment. By utilizing sugarcane bagasse, the research adhered to the principles of reuse, recycling, and material recovery, transforming solid waste into a value-added material for effective wastewater management. Additionally, the synthesis of nZVI using Sal leaf extract as a natural reducing agent instead of conventional chemical agents was based on the principle of green reduction. These innovative practices are pivotal for achieving sustainability goals and advancing the circular economy. Furthermore, a comprehensive cost analysis revealed that this method of treating antibiotic-laden wastewater is cost-effective under optimal conditions, highlighting its potential for practical application on a larger scale. The study not only addresses environmental pollution but also provides a sustainable and economically viable solution for wastewater management, promoting broader implementation in real-world scenarios.

### Limitations of the study

While this process is effective and eco-friendly, it has some limitations. Zeta potential analysis of the nanocomposites could not be performed, preventing a detailed understanding of particle stability. Quenching experiments to analyze adsorption patterns were also not conducted. Additionally, the degradation efficiency depends on environmental conditions, requiring re-optimization for each antibiotic, which adds complexity. Industrial wastewater, often containing multiple antibiotics, may require further optimization for simultaneous treatment. These factors highlight the need for additional research to ensure broader applicability and efficiency under varying real-world conditions.

## Resource availability

### Lead contact

Requests for further information and resources should be directed to and will be fulfilled by the lead contact, Dr. Sukalyan Chakraborty (sukalyanchakraborty@bitmesra.ac.in).

### Material availability

The nano-composite material synthesized in this study can be synthesized based on the method detailed in the STAR Methods section.

### Data and code availability

• **Data:** All data generated or analyzed during this study are included in the article and supplementary tables and figures.
• **Code:** No program code has been used in this study.
• Any additional information required to reanalyze the data reported in this article will be available from the lead contact upon request.

## Acknowledgments

The authors would like to acknowledge Birla Institute of Technology, Mesra, for providing financial support for conducting the study through the Institute Research Fellowship to Aditya Kumar Jha (Award no: GO/Estb/Ph.D/2018-19/2184. Registration no: 170406) and lab funds. The authors are thankful to the Indian Institute of Technology, Bombay, and the Central Instrumentation Facility of Birla Institute of Technology, Mesra, for facilitating the characterization part of the study.

## Author contributions

AKJ: Conceptualization, data curation, formal analysis, visualization, and writing - original draft. SC: Conceptualization, validation, and writing - review and editing. JKB: Conceptualization and writing - review and editing.

## Declaration of interests

One of the co-authors, Jayanta Kumar Biswas, is the Guest Editor of the special issue in which this article will appear but was not involved in the editorial workflow or peer review process.

## STAR★Methods

### Key resources table

**Table:** 

REAGENT or RESOURCE	SOURCE	IDENTIFIER
Tetracycline	Alfa Aesar (Great Britain)	CAS No: 60-54-8
Ciprofloxacin	Alfa Aesar (China)	CAS No: 85721-33-1
Sulfuric acid	Rankem (India)	CAS No: 7664-93-9
Sodium hydroxide	Rankem (India)	CAS No: 1310-73-2
Ferrocene	SRL	CAS No: 102-54-5

**Software and Algorithms**		

ImageJ	https://imagej.net/ij/download.html	ImageJ 1.54G

### Experimental model and study participant details

#### Ethic statement

No animals were used in this study.

### Method details

#### Synthesis of GO-nZVI composite

First, to synthesize nZVI, the *Shorea robusta* (Sal) leaf extract was used as the reducing agent, while the FeSO_4_.7H_2_O was used as the precursor. To prepare the leaf extract, the Sal leaves were collected, cleaned with deionized water, and blot-dried to remove the dust particles. After weighing the leaves, they were crushed using a mortar and pestle and mixed with water until a smooth paste was formed. The resulting mixture was then diluted with water in a ratio of 10:1 (i.e., 10 mL of water per 1 g of leaf) to achieve the final volume. The mixture was then passed through a membrane filter to obtain the leaf extract, which was stored at 4°C for further application.

Graphene oxide was synthesized using the slightly modified protocol of Somanathan et al..^39^ Sugarcane bagasse, a solid waste produced when the juice is extracted from sugarcane, was collected, rinsed twice with deionized water, blotted, and oven-dried at 80°C to eliminate the excess moisture. The dried biomass underwent grinding and then passed through a sieve of 63 microns to avoid larger chunks. The sugarcane bagasse was combined with ferrocene in a proportion of 6:1, loaded in a crucible, and placed in a muffle furnace at 300°C under normal atmospheric conditions for 20 min to obtain GO.

To synthesize the GO-nZVI composite, synthesized GO powder was mixed with 0.01M FeSO_4_.7H_2_O solution in Erlenmeyer flasks and mixed using a magnetic stirrer at 200 rpm for 30 min. Sal leaf extract of the same volume as FeSO_4_.7H_2_O was then added dropwise into the Erlenmeyer flask. The mixture was then placed in an orbital shaker at 200 rpm, at 27°C, left overnight and then filtered using a membrane with a pore size of 45 μm to isolate the composite. The composite was oven-dried and stored in a desiccator (Figure S4). The optimal ratio of FeSO_4_.7H_2_O and Sal leaf extract to a fixed weight of GO was determined based on the degradation efficiency against 20 mgL^-1^ CP solution. The best combination ratio corresponded to the best degradation efficiency. The volume of FeSO_4_.7H_2_O and Sal leaf extract varied from 20 mL to 140 mL, each with an interval of 20 mL to 0.1 g of GO powder. The composite with the highest CP removal efficiency was selected for further study (Figure S1).

#### Characterization of GO-nZVI composite

The morphology and particle size of the formed GO-nZVI composite were characterized by Field emission scanning electron microscope (FESEM: Sigma 300, Carl Zeiss, Germany) and high-resolution transmission electron microscope (HRTEM: JEM 2100F 200KV, Jeol, Japan). Elemental composition was determined using energy-dispersive X-ray spectroscopy (EDX: JOEL JSM-6390LV, Jeol Japan). X-ray diffraction (XRD: Smart Lab 9KW, Rigaku, Japan)was used to study the phase character of the synthesized GO. Fourier transform infrared spectroscopy (FTIR: IR Prestige 2, Shimadzu Corporation, Japan) of the synthesized material was conducted in the range of 4000–400 cm^−1^ to know the probable functional groups responsible for TET and CP degradation. Raman Spectroscopy (Renishaw inVia, UK, excitation wavelength: 514 nm) was used to study the microstructure synthesis of GO, where GO and impurities in its structure can be studied using its characteristic D and G band.^15^

#### Photocatalytic degradation of antibiotics using GO-nZVI composite

Photocatalytic degradation of TET and CP with GO-nZVI composite was investigated in a triplicate batch culture setup under varying experimental conditions. Antibiotic solutions (50 mL) were taken separately in a 100 mL Erlenmeyer flask, and GO-nZVI composite was added to it under stirring conditions. Residual concentrations of TET and CP were recorded at 357 nm and 277 nm using UV-visible Spectroscope (Shimadzu UV-1800, Cole-Parmer, Japan), respectively.^40^ The degradation efficiency was determined using Equation 1.^41^

(Equation 1) $$\text{Photocatalytic}-\text{degradation}\;\text{efficiency}\;\left(\%\right)=\left(\frac{C_{0}-C_{t}}{C_{0}}\right)*100$$

#### Reagents used

For optimum degradation of TET and CP, the following conditions were varied:

1) The effects of light on the degradation of TET and CP were investigated under the dark, UV light and Sunlight (750–850 Wm^-2^). A 40 Watts UV lamp provided UV light in a closed box measuring 30 × 30 × 45 cm. 2) TET and CP concentrations varied as 5, 10, 15, 20, 25, 30 and 35 mgL^-1^. 3) The dose of GO-nZVI composite was varied as 0.05, 0.10, 0.15, 0.20, 0.25, and 0.30 gmL^−1^. 4) The initial pH of the solution was varied as 2, 4, 6, 8, and 10 using sulfuric acid(0.1M) and sodium hydroxide (0.1 M). 5) The stirring was varied between 100 and 500 rpm. The optimum time for the photocatalytic degradation of TET and CP by GO-nZVI composite was determined by studying the degradation every 10 min up to 80 and 100 min, respectively (Figure S5). Mass spectrometry (MS: Ultimate 3000, LTQ XL, Thermo Scientific, USA) was used to identify the degraded products in the antibiotic solution in the range of 50–500 m/z.

A bioassay was performed using a cup-plate method to check the toxicity of the photo-catalytically degraded residual antibiotics. Initially, bacterial strains were isolated from water samples of River Subarnarekha (23.4073919° N, 85.4397186° E) in nutrient agar plates after serial dilution. Axenic cultures of four bacterial strains with distinct colony patterns were selected for the study, and they were sub-cultured in nutrient broth. Each bacterial strain was grown in nutrient agar plates and tested for toxicity in the presence of the degraded antibiotics applied in wells under incubation at 370°C for 24 h. Zone of inhibition was noted as an indicator of toxicity due to the degraded antibiotics.^42^^,^^43^

#### Degradation kinetics

To gain a precise understanding of the possible mechanism of the synthesised nZVI on the photocatalytic degradation of TET and CP, the time-dependent degradation data was meticulously fitted to commonly used Langmuir-Hinshelwood kinetic models, including pseudo-first and pseudo-second-order reaction models, which were meticulously calculated using Equation 2 and Equation 3 respectively.^44^^,^^45^^,^^46^

(Equation 2) $$\ln\;\frac{C}{C_{0}}=-K_{1\;}t$$

(Equation 3) $$\frac{1}{C}=\frac{1}{C_{0}}+K_{2}t$$

#### Cost analysis

A cost analysis was performed to study the economic feasibility of applying GO-nZVI composite-mediated degradation of TET and CP. First, the cost of GO-nZVI composite synthesis was determined, including the cost of raw materials, chemicals, and electricity. Using the required doses of these photocatalysts for the degradation of TET and CP, the cost of their degradation was calculated separately.

### Quantification and statistical analysis

ImageJ 1.54G software (https://imagej.net/ij/download.html) was used to analyze the images of the GO-nZVI composites obtained by the HRTEM. The results have been presented in the results and discussion section of the GO-nZVI composite.

VOSviewer version 1.6.20 was used for the bibliometric analysis after procuring the data from the link: photocatalytic degradation antibiotics nano (Topic) – 276 – Web of Science Core Collection. The result has been presented in the Figure S3.

OriginPro 2024b (64bit) 10.1.5.132 (Learning edition) was used to construct the optimization and the degradation graphs.

## References

[1] Le T.-H., Ng C., Tran N.H., Chen H., Gin K.Y.-H. (2018). Removal of antibiotic residues, antibiotic resistant bacteria and antibiotic resistance genes in municipal wastewater by membrane bioreactor systems. Water Res..

[2] Ben Y., Fu C., Hu M., Liu L., Wong M.H., Zheng C. (2019). Human health risk assessment of antibiotic resistance associated with antibiotic residues in the environment: A review. Environ. Res..

[3] Al Omari S., Al Mir H., Wrayde S., Merhabi S., Dhaybi I., Jamal S., Chahine M., Bayaa R., Tourba F., Tantawi H. (2019). First Lebanese Antibiotic Awareness Week campaign: knowledge, attitudes and practices towards antibiotics. J. Hosp. Infect..

[4] Ezzariai A., Hafidi M., Khadra A., Aemig Q., El Fels L., Barret M., Merlina G., Patureau D., Pinelli E. (2018). Human and veterinary antibiotics during composting of sludge or manure: Global perspectives on persistence, degradation, and resistance genes. J. Hazard Mater..

[5] Tan X., Liu Y., Zeng G., Wang X., Hu X., Gu Y., Yang Z. (2015). Application of biochar for the removal of pollutants from aqueous solutions. Chemosphere.

[6] Zhang L., Qin S., Shen L., Li S., Cui J., Liu Y. (2020). Bioaccumulation, trophic transfer, and human health risk of quinolones antibiotics in the benthic food web from a macrophyte-dominated shallow lake, North China. Sci. Total Environ..

[7] Jha A.K., Chakraborty S. (2020). Photocatalytic degradation of Congo Red under UV irradiation by zero valent iron nano particles (nZVI) synthesized using Shorea robusta (Sal) leaf extract. Water Sci. Technol..

[8] Jha A.K., Chakraborty S. (2023). Photocatalytic degradation of tetracycline and ciprofloxacin antibiotic residues in aqueous phase by biosynthesized nZVI using Sal ( Shorea robusta ) leaf extract. J. Water Supply Res. Technol. - Aqua.

[9] Yousefinia S., Sohrabi M.R., Motiee F., Davallo M. (2023). Enhanced simultaneous removal of direct red 81 and bisphenol A from aqueous media by coupling nano zerovalent iron (nZVI) particles with graphene oxide and copper: Isotherm and kinetic adsorption studies. Mater. Chem. Phys..

[10] Qiao D., Li Z., Duan J., He X. (2020). Adsorption and photocatalytic degradation mechanism of magnetic graphene oxide/ZnO nanocomposites for tetracycline contaminants. Chem. Eng. J..

[11] Aydin S., Aydin M.E., Ulvi A., Kilic H. (2019). Antibiotics in hospital effluents: occurrence, contribution to urban wastewater, removal in a wastewater treatment plant, and environmental risk assessment. Environ. Sci. Pollut. Res. Int..

[12] Bhagat C., Kumar M., Tyagi V.K., Mohapatra P.K. (2020). Proclivities for prevalence and treatment of antibiotics in the ambient water: a review. Npj Clean Water.

[13] Chen F., Wu X.-L., Yang L., Chen C., Lin H., Chen J. (2020). Efficient degradation and mineralization of antibiotics via heterogeneous activation of peroxymonosulfate by using graphene supported single-atom Cu catalyst. Chem. Eng. J..

[14] Govindan K., Suresh A.K., Sakthivel T., Murugesan K., Mohan R., Gunasekaran V., Jang A. (2019). Effect of peroxomonosulfate, peroxodisulfate and hydrogen peroxide on graphene oxide photocatalytic performances in methyl orange dye degradation. Chemosphere.

[15] Cai J., Zhang Y. (2022). Enhanced degradation of bisphenol S by persulfate activated with sulfide-modified nanoscale zerovalent iron. Environ. Sci. Pollut. Res. Int..

[16] Boonruam P., Soisuwan S., Wattanachai P., Morillas H., Upasen S. (2021). Solvent Effect On Zerovalent Iron Nanoparticles (nZVI) Preparation And Its Thermal Oxidation Characteristic. ASEAN Engineering Journal.

[17] Zhang Y., Zhao L., Yang Y., Sun P. (2018). Degradation of the antibiotic ornidazole in aqueous solution by using nanoscale zerovalent iron particles: kinetics, mechanism, and degradation pathway. RSC Adv..

[18] Zhan W., Gao L., Fu X., Siyal S.H., Sui G., Yang X. (2019). Green synthesis of amino-functionalized carbon nanotube-graphene hybrid aerogels for high performance heavy metal ions removal. Appl. Surf. Sci..

[19] Pei S., Wei Q., Huang K., Cheng H.-M., Ren W. (2018). Green synthesis of graphene oxide by seconds timescale water electrolytic oxidation. Nat. Commun..

[20] Liu Y., Zhang X., Zhou Y., Ma H., Cheng X., Wei L., Hou Z. (2022). MFO@NZVI/hydrogel for sulfasalazine degradation: Performance, mechanism and degradation pathway. Separ. Purif. Technol..

[21] Wei Q., Li H., Guo Y., Gao C., Li R., Zhou A., Wang S., Yue X. (2023). Performance and mechanism study of g-C3N4/rGO heterojunction enhanced NO3− reduction by nZVI under visible light irradiation. J. Alloys Compd..

[22] Shaikh W.A., Chakraborty S., Kumar A., Biswas J.K., Jha A.K., Bhattacharya T., Vithanage M., Ansar S., Hossain N. (2023). Tailor-made biochar-based nanocomposite for enhancing aqueous phase antibiotic removal. J. Water Proc. Eng..

[23] Song M., Hu X., Gu T., Zhang W.x., Deng Z. (2022). Nanocelluloses affixed nanoscale Zerovalent iron (nZVI) for nickel removal: Synthesis, characterization and mechanisms. J. Environ. Chem. Eng..

[24] Chamanehpour E., Sayadi M.H., &Hajiani M. (2023). Metal-organic framework coordinated with g-C3N4 and metal ions for boosting photocatalytic H2 production under sunlight. J. Photochem. Photobiol. Chem..

[25] Gupta B., Gupta A.K., Tiwary C.S., Ghosal P.S. (2020). A multivariate modeling and experimental realization of photocatalytic system of engineered S–C3N4/ZnO hybrid for ciprofloxacin removal: Influencing factors and degradation pathways. Environ. Res..

[26] Moradi S., Sobhgol S.A., Hayati F., Isari A.A., Kakavandi B., Bashardoust P., Anvaripour B. (2020). Performance and reaction mechanism of MgO/ZnO/Graphene ternary nanocomposite in coupling with LED and ultrasound waves for the degradation of sulfamethoxazole and pharmaceutical wastewater. Separ. Purif. Technol..

[27] Rezaei A., Rezaei M.R., Sayadi M.H. (2021). Enhanced 3,5-dimethylphenol photodegradation via adsorption-photocatalysis synergy using FSTRG nanohybrid catalyst. J. Mol. Liq..

[28] Ren F., Zhu W., Zhao J., Liu H., Zhang X., Zhang H., Zhu H., Peng Y., Wang B. (2020). Nitrogen-doped graphene oxide aerogel anchored with spinel CoFe2O4 nanoparticles for rapid degradation of tetracycline. Separ. Purif. Technol..

[29] Fu Y., Peng L., Zeng Q., Yang Y., Song H., Shao J., Liu S., Gu J. (2015). High efficient removal of tetracycline from solution by degradation and flocculation with nanoscale zerovalent iron. Chem. Eng. J..

[30] Huang Y., Nengzi L.c., Zhang X., Gou J., Gao Y., Zhu G., Cheng Q., Cheng X. (2020). Catalytic degradation of Ciprofloxacin by magnetic CuS/Fe2O3/Mn2O3 nanocomposite activated peroxymonosulfate: Influence factors, degradation pathways and reaction mechanism. Chem. Eng. J..

[31] Xing R., He J., Hao P., Zhou W. (2020). Graphene oxide-supported nanoscale zerovalent iron composites for the removal of atrazine from aqueous solution. Colloids Surf. A Physicochem. Eng. Asp..

[32] Guerrero-Fajardo C.A., Giraldo L., Moreno-Piraján J.C. (2020). Preparation and Characterization of Graphene Oxide for Pb(II) and Zn(II) Ions Adsorption from Aqueous Solution: Experimental, Thermodynamic and Kinetic Study. Nanomaterials.

[33] Tarekegn M.M., Hiruy A.M., Dekebo A.H. (2021). Nano zero valent iron (nZVI) particles for the removal of heavy metals (Cd 2+ , Cu 2+ and Pb 2+ ) from aqueous solutions. RSC Adv..

[34] Manea Y.K., Khan A.M., Wani A.A., Qashqoosh M.T., Shahadat M., Salem M.A. (2021). Hydrothermally synthesized mesoporous CS-g-PA@TSM functional nanocomposite for efficient photocatalytic degradation of Ciprofloxacin and treatment of metal ions. J. Mol. Liq..

[35] Ahmadpour N., Sayadi M.H., Sobhani S., Hajiani M. (2020). A potential natural solar light active photocatalyst using magnetic ZnFe2O4 @ TiO2/Cu nanocomposite as a high performance and recyclable platform for degradation of naproxen from aqueous solution. J. Clean. Prod..

[36] Ahmadpour N., Sayadi M.H., Sobhani S., Hajiani M. (2020). Photocatalytic degradation of model pharmaceutical pollutant by novel magnetic TiO2@ZnFe2O4/Pd nanocomposite with enhanced photocatalytic activity and stability under solar light irradiation. J. Environ. Manag..

[37] Alam S.N., Sharma N., Kumar L. (2017). Synthesis of Graphene Oxide (GO) by Modified Hummers Method and Its Thermal Reduction to Obtain Reduced Graphene Oxide (rGO). Graphene.

[38] Orimolade B.O., Zwane B.N., Koiki B.A., Tshwenya L., Peleyeju G.M., Mabuba N., Zhou M., Arotiba O.A. (2020). Solar photoelectrocatalytic degradation of Ciprofloxacin at a FTO/BiVO4/MnO2 anode: Kinetics, intermediate products and degradation pathway studies. J. Environ. Chem. Eng..

[39] Somanathan T., Prasad K., Ostrikov K.K., Saravanan A., Krishna V.M. (2015). Graphene Oxide Synthesis from Agro Waste. Nanomaterials.

[40] Che H., Che G., Jiang E., Liu C., Dong H., Li C. (2018). A novel Z-Scheme CdS/Bi3O4Cl heterostructure for photocatalytic degradation of antibiotics: Mineralization activity, degradation pathways and mechanism insight. J. Taiwan Inst. Chem. Eng..

[41] Selvamani P.S., Vijaya J.J., Kennedy L.J., Mustafa A., Bououdina M., Sophia P.J., Ramalingam R.J. (2021). Synergic effect of Cu2O/MoS2/rGO for the sonophotocatalytic degradation of tetracycline and ciprofloxacin antibiotics. Ceram. Int..

[42] Abdollahzadeh S., Sayadi M.H., Shekari H. (2023). Synthesis of biodegradable antibacterial nanocomposite (metal–organic frameworks supported by chitosan and graphene oxide) with high stability and photocatalytic activities. Inorg. Chem. Commun..

[43] Bhuin A., Udayakumar S., Gopalarethinam J., Mukherjee D., Girigoswami K., Ponraj C., Sarkar S. (2024). Photocatalytic degradation of antibiotics and antimicrobial and anticancer activities of two-dimensional ZnO nanosheets. Sci. Rep..

[44] Sundararajan M., Sailaja V., John Kennedy L., Judith Vijaya J. (2017). Photocatalytic degradation of rhodamine B under visible light using nanostructured zinc doped cobalt ferrite: kinetics and mechanism. Ceram. Int..

[45] Bagtache R., Abdmeziem K., Dib K., Trari M. (2018). Synthesis and photoelectrochemical characterization of KZn_2_(HPO_4_)PO_4_: application to rhodamine B photodegradation under solar light. Int. J. Environ. Sci. Technol..

[46] Chakraborty S., Jha A.K. (2021). Photocatalytic degradation of Ciprofloxacin by bagasse derived graphene oxide and toxicity test of the degraded products through microbiological assay. Int. J. Environ. Pollut..

