# Supplementary material for: Green synthesis of low-cost graphene oxide-nano zerovalent iron composite from solid waste for photocatalytic removal of antibiotics
Source: iScience. 2024 Nov 28;27(12):111486. doi: 10.1016/j.isci.2024.111486 (PMC11700629; doi:10.1016/j.isci.2024.111486)
Supplement: Document S1. Figures S1–S6, Tables S1 and S2 [file mmc1.pdf]

iScience, Volume *27*

Supplemental information

Green synthesis of low-cost graphene oxide-nano zerovalent iron composite from solid waste for photocatalytic removal of antibiotics

Aditya Kumar Jha, Sukalyan Chakraborty, and Jayanta Kumar Biswas

Supplementary information

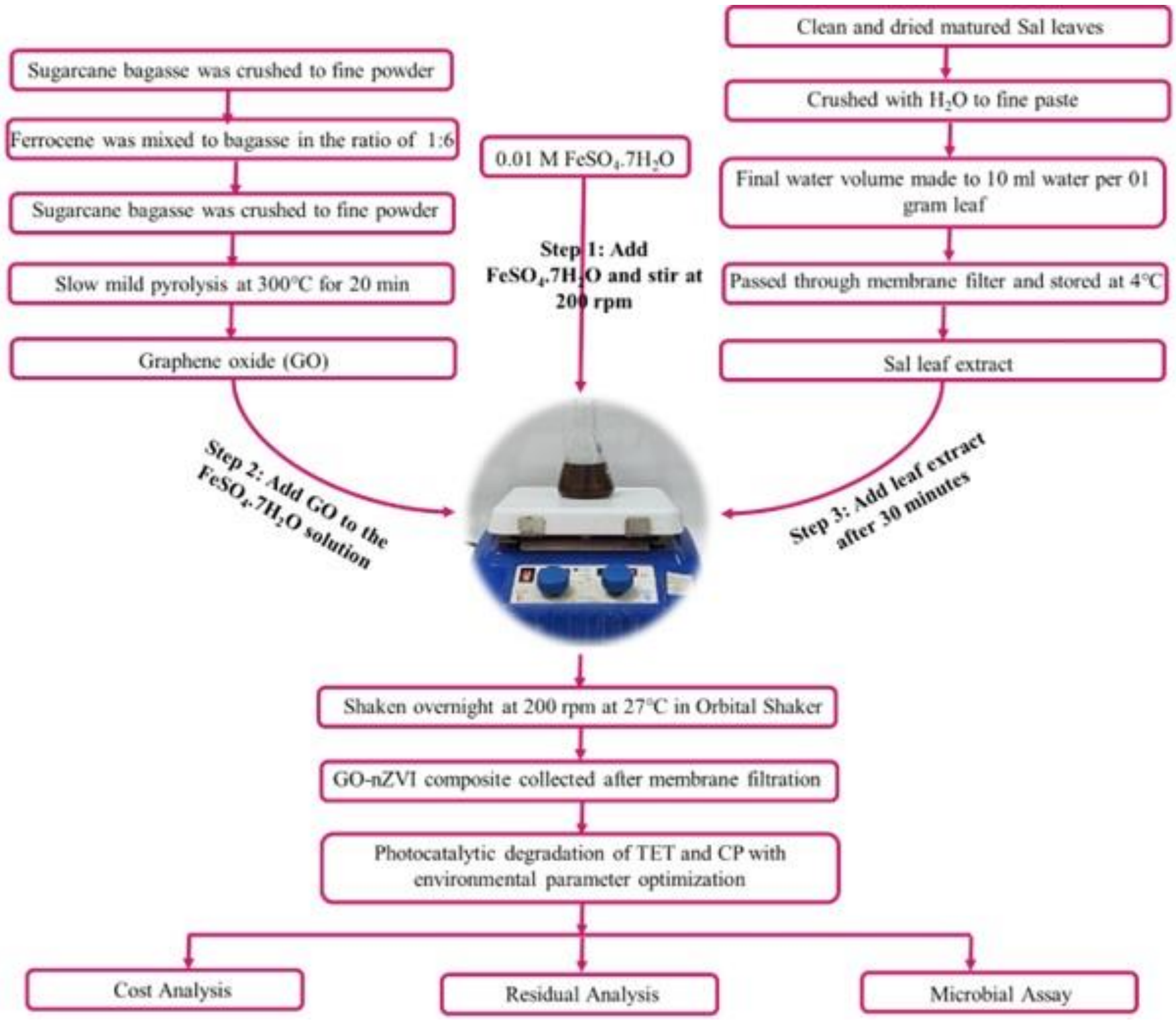

Supplementary Figure 1: Scheme for green synthesis of GO, nZVI and GO-nZVI composite using Sal (Shorea robusta) leaf extract and removal of antibiotics (tetracycline and Ciprofloxacin) from contaminated water.

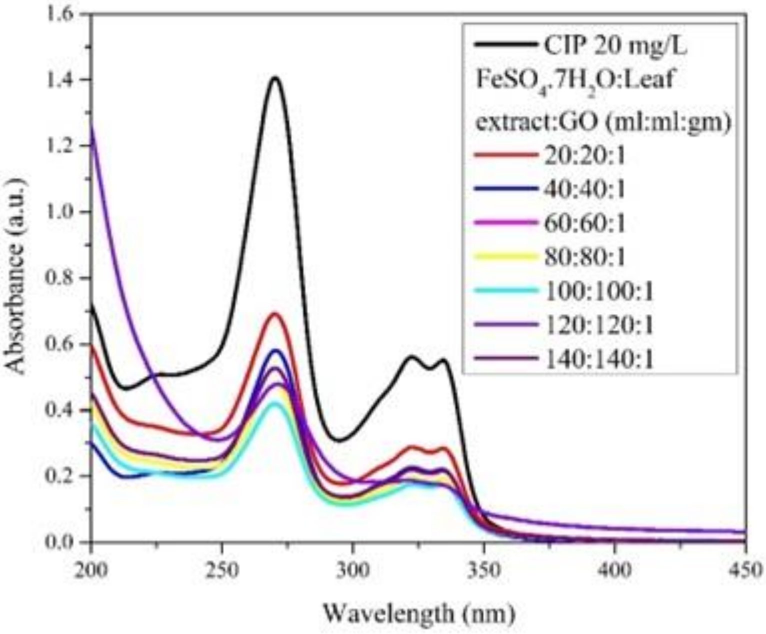

Supplementary Figure 2: Optimization of GO-nZVI composite.

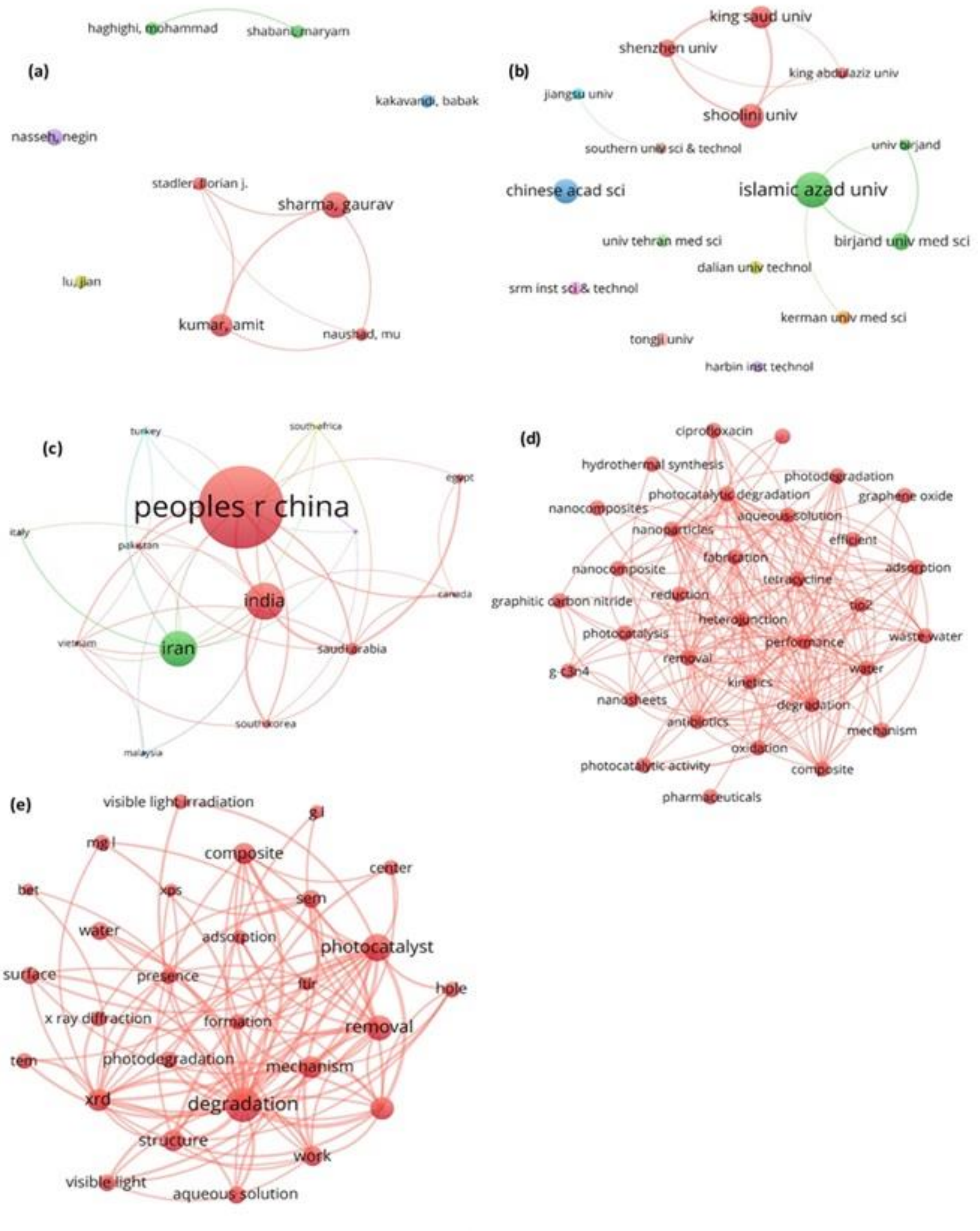

Supplementary Figure 3:Top (a) authors, (b) organizations, (c) countries, (d) All keyword maps, and (e) Term maps.

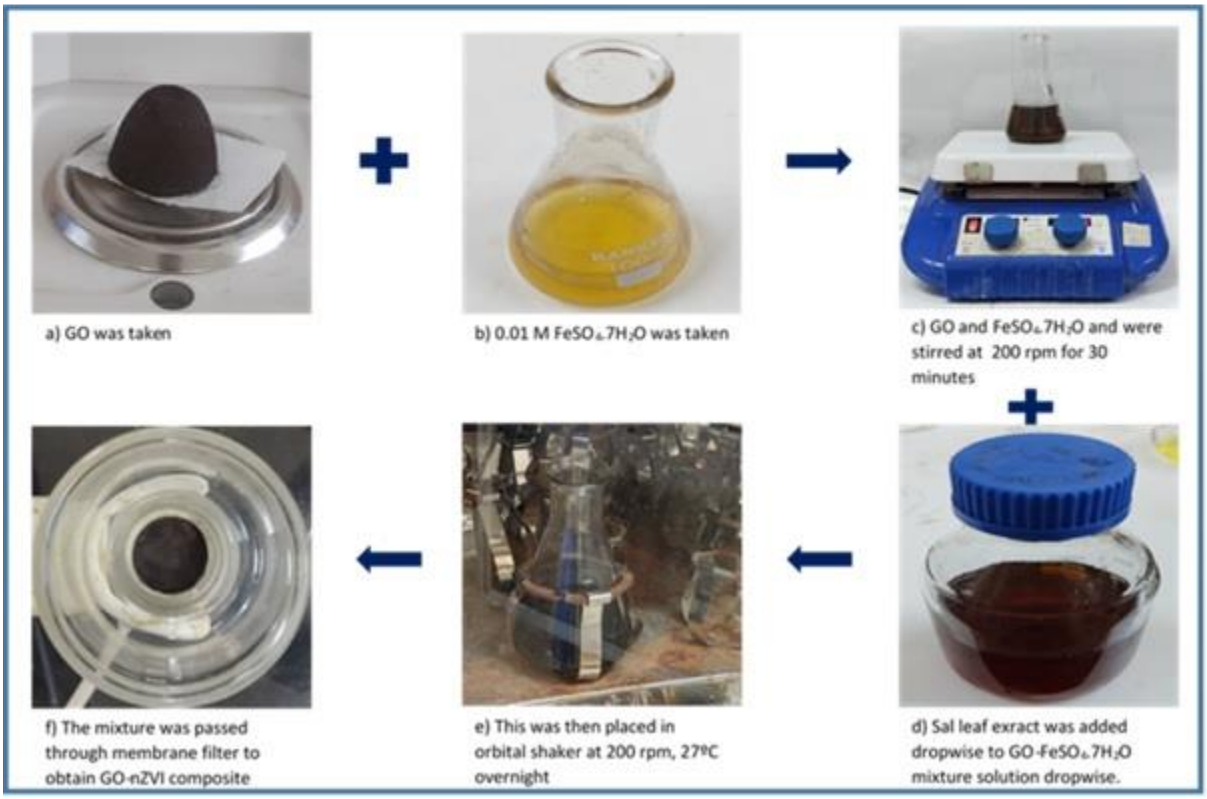

Supplementary Figure 4: Schematic diagram for the synthesis of GO-nZVI composite.

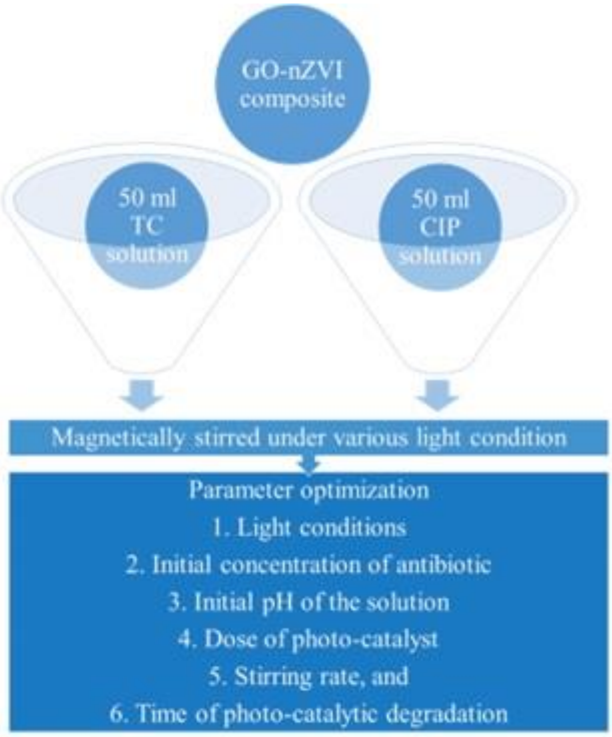

Supplementary Figure 5: Schematic diagram for the degradation of TET and TC.

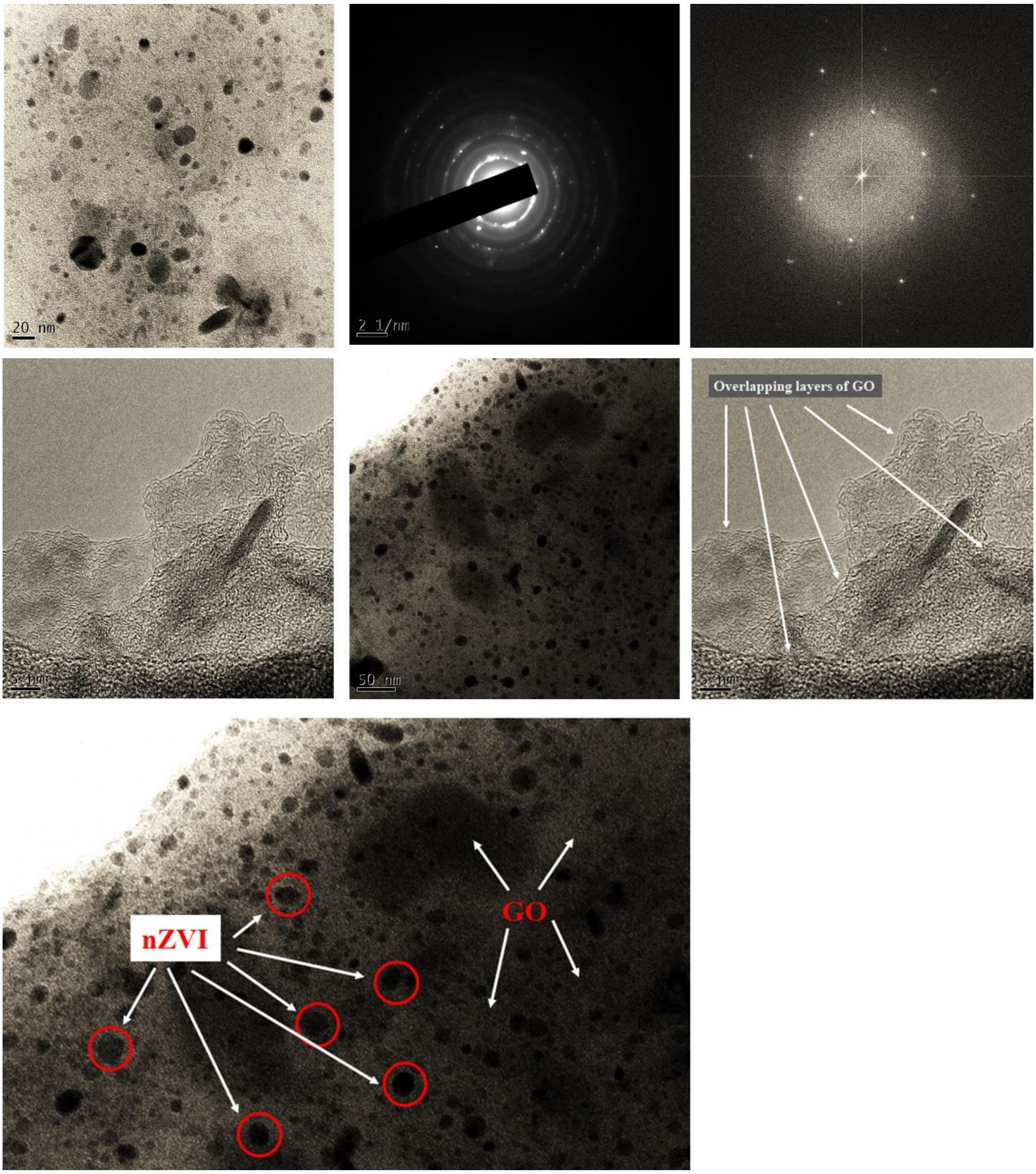

Supplementary Figure 6: Mapped images of HRTEM.

Supplementary table 1: List of most cited articles onphotocatalytic degradation antibiotics by nano-materials

Rank	Title	Author	Journal	Citation
1	"Rational design on 3D hierarchical bismuth oxyiodides via in situ self-template phase transformation and phase-junction construction for optimizing photocatalysis against diverse contaminants	(H. Huang et al., 2017)	Applied Catalysis B-Environmental	253
2	In-situ electro-generation and activation of hydrogen peroxide using a CuFeNLDH-CNTs modified graphite cathode for degradation of cefazolin	(Ghasemi et al., 2020)	Journal of Environmental Management	148
3	Aqueous tetracycline degradation by non-thermal plasma combined with nano-TiO_2	(He et al., 2014)	Chemical Engineering Journal	143
4	Enhanced activity of clinoptilolite-supported hybridized PbS-CdS semiconductors for the photocatalytic degradation of a mixture of tetracycline and cephalexin aqueous solution	(Azimi & Nezamzadeh -Ejhieh, 2015)	Journal of Molecular Catalysis A-Chemical	140
5	Fabrication of g-C_3N_4/Ti_3C_2 composite and its visible-light photocatalytic capability for CProfloxacin degradation	(N. Liu et al., 2019)	Separation And Purification Technology	131
6	N, Cu co-doped TiO_2@functionalized SWCNT photocatalyst coupled with ultrasound and visible-light: An effective sono-photocatalysis process for pharmaceutical wastewaters treatment	(Isari et al., 2020)	Chemical Engineering Journal	116
7	Fabrication and characterization of trimetallic nano-photocatalyst for remediation of ampicillin antibiotic	(Sharma et al., 2018)	Journal of Molecular Liquids	115
8	Photocatalytic degradation of CProfloxacin antibiotic by TiO_2 nanoparticles immobilized on a glass plate	(Malakootian et al., 2020)	Chemical Engineering Communications	110
9	Impact of post-processing modes of precursor on adsorption and photocatalytic capability of mesoporous TiO_2nanocrystallite aggregates towards CProfloxacin removal	(Gan et al., 2018)	Chemical Engineering Journal	109
10	Tetracycline degradation in aquatic environment by highly porous MnO_2 nanosheet assembly"	(Mahamallik et al., 2015)	Chemical Engineering Journal	109

Supplementary table 2: List of most prominent authors, organizations, countries, funding agencies and topics.

**Authors**	**Articles**	**Organization**	**Articles**	**Country**	**Articles**
Kumar, Amit	7	Islamic Azad University	17	China	125
Naushad, Mohammad	6	Chinese Academy of Sciences	12	India	57
Sharma, Gaurav	6	Egyptian Knowledge Bank	12	Iran	56
Nasseh, Negin	5	Shoolini University	11	Saudi Arabia	20
Stadler, Florian J.	4	King Saud University	10	Egypt	13
**Publisher**	**Articles**	**Journal**	**Articles**	**Research Area**	**Articles**
Elsevier	171	Chemical Engineering Journal	21	Chemistry	99
Springer Nature	32	Chemosphere	15	Engineering	99
Royal Society of Chemistry	14	Separation and Purification Technology	14	Environmental Sciences Ecology	64
American Chemical Society	8	Desalination and Water Treatment	9	Materials Science	54
Desalination Publication	8	Journal of Alloys and Compounds	8	Physics	2
**Funding Agencies**	**Articles**	**Meso-Citation Topic**	**Articles**	**Micro-Citation Topic**	**Articles**
National Natural Science Foundation of China	89	Photocatalysts	160	Photocatalysis	154
Fundamental Research Funds for The Central Universities, China	11	Water Treatment	52	Electrocoagulation	35
China Postdoctoral Science Foundation	10	Nanoparticles	17	Silver Nanoparticles	14
Department of Science Technology, India	8	Herbicides, Pesticides & Ground Poisoning	12	Adsorption	11
Natural Science Foundation of Jiangsu Province, China	7	Inorganic & Nuclear Chemistry	5	Bisphenol A	11

Data availability statements: Data supporting this study are included within the article and/or supporting material
